# Emergence of Persistent Infection due to Heterogeneity

**DOI:** 10.1038/srep41582

**Published:** 2017-02-01

**Authors:** Vidit Agrawal, Promit Moitra, Sudeshna Sinha

**Affiliations:** 1 Deprtment of Physics, University of Arkansas, Fayetteville, Arkansas AR 72701, USA; 2 Indian Institute of Science Education and Research (IISER) Mohali, Knowledge City, SAS Nagar, Sector 81, Manauli PO 140 306, Punjab, India

## Abstract

We explore the emergence of persistent infection in a closed region where the disease progression of the individuals is given by the SIRS model, with an individual becoming infected on contact with another infected individual. We investigate the persistence of contagion qualitatively and quantitatively, under increasing heterogeneity in the partitioning of the population into different disease compartments, as well as increasing heterogeneity in the phases of the disease among individuals within a compartment. We observe that when the initial population is uniform, consisting of individuals at the same stage of disease progression, infection arising from a contagious seed does not persist. However when the initial population consists of randomly distributed refractory and susceptible individuals, a single source of infection can lead to sustained infection in the population, as heterogeneity facilitates the de-synchronization of the phases in the disease cycle of the individuals. We also show how the average size of the window of persistence of infection depends on the degree of heterogeneity in the initial composition of the population. In particular, we show that the infection eventually dies out when the entire initial population is susceptible, while even a few susceptibles among an heterogeneous refractory population gives rise to a large persistent infected set.

How a disease spreads in a population is a question of much interest and relevance, and consequently has been extensively investigated over the years^1^,^2^,^3^. Different classes of models mimicking infection spread are obtained by exploring different models of disease progression at the local/individual level. These may be simple disease cycles, that end in fatality or permanent immunity, or they may be diseases that have more complicated progression, including refractory periods where immunity is temporary. Further, experimental observations, such as of the spread of measles in Iceland, which being isolated provides a “natural laboratory” for the study of epidemics spreading, indicated that the spatial element is essential in constructing any theory for valid predictions^3^. So a variety of models have been propounded, taking into account different properties of the spatial domain in which the infection spreads.

A wide class of models focus principally on infection transmission, keeping the individual disease progression at the local level simple. For instance, there are models based on local transmission of infection, with the infection being ultimately fatal^4^. The probability of infection from the infected host (transmissibility) is found to be the crucial parameter in such host-pathogen models, and the pathogen must have a minimum transmissibility in order to propagate, with the host driven to extinction if it exceeds a certain transmissibility. Further simple models of disease transmission on small-world networks has been investigated^5^, varying the probability of infection by a disease and/or the probability of its transmission. Such models display epidemic behavior when the infection or transmission probability is above a threshold, analogous to percolation thresholds. Similar studies on scale-free networks, with the disease cycle ending with permanent immunity shows that no long-term infection can be maintained as the number of susceptibles decline as the epidemic spreads^6^. Studies of disease models where the individuals become susceptible again, right after the infective period, shows that scale-free networks are more prone to spreading and persistance of infection, while exponential networks have an epidemic threshold above which the infection becomes persistent in time^7^.

Another class of models investigates more complicated disease progression, such as diseases with temporary immunity. For instance in ref. 8, for representative parameters the model exhibits expanding circular waves of infection, some of which are generated by unusual ‘pacemaker centres’. When infected individuals recover, the interior of the growing wave boundary becomes a fresh pool of susceptible individuals. At the end of the cycle, a distant infectee short-cuts through the network to reinfect the waves focal pacemaker, enabling it to perpetuate. The results from this study suggest that both the temporary immunity and the social structure have equal influence on the existence of periodicity in the disease outbursts, and small-world connectivity was seen to lead to persistent infection. A model with a similar disease progression has been studied, with infection rate being a control parameter^9^. By using deterministic (mean field) equations to describe the temporal evolution of the disease, it was found that the epidemics will be persistent when the control parameter is bounded in a certain range, and if the infection rate is sufficiently large, too many susceptibles get infected at the same time and therefore die simultaneously, leading to extinction.

Similar investigations of a generic model of excitable media with increasing density of random long-range connections^10^, reported the existence of two qualitatively different regimes of self-sustained pattern formation. Starting from nearest neighbor coupling, as the random links were increased, dynamically different results were observed. Below a lower critical probability, the state of the system after an initial transient period was characterized by self-sustaining single or multiple spiral waves. Second, at the critcial probability, the spiral wave mode was suppressed and the system underwent a transition to periodic activity. Third, when the value of probability was increased above a system-size-dependent upper critical probability, the self-sustained activity ceased. This occured due to the spiral waves being primarily created by shortcut-induced excitations. So a very large number of shortcut connections guaranteed almost simultaneous spread of excitation to nearly all cells, as a result of which the dynamics of the system tended to ‘burn out’ after a transient. Several other studies on small-world networks also showed the emergence of epidemic outbreaks for sufficiently large number of random connections^11^,^12^. Further it was shown that time-varying networks yield epidemics more readily than static networks^13^.

Lattice-based models for dealing with spatially-distributed host population, have also been explored. For instance, ref. 14 yield results suggestive of self-organised criticality. By attaining a critical prevalence, the disease appears to reach a state where small fluctuations have the potential to induce cascades of infection on a wide range of time and length scales. This, in effect, maximises possibility of persistence by ensuring all spatial and temporal scales are accessed. In the context of persistence, another very interesting study^15^ focuses on the local aspects of inhomogeneity in a spatially homogeneous environment. In this work, the excitable media is characterized by a globally stable equilibrium state, and also by a threshold mechanism which produces a large amplitude response to a sufficiently large stimulus. It was shown that spatial inhomogeneities in such an excitable media tended to produce spatial patterns which oscillated periodically in time.

Lastly, a large class of models consider the disease progression in homogeneously well-mixed populations, where encounters are random, using an approach inspired by mass action kinetics, resulting in a sets of ordinary differential equations^16^,^17^,^18^,^19^. The advantage of these commonly used models based on differential equations (or difference equations^18^) is that they are most often analytically tractable, and can yield asymptotic stability regions for the equilibrium points. However, certain features are lost, as the homogeneity of a large population is the underlying assumption in these models.

In this work we will explore the following crucial question, that has not seen much focus yet: *what population compositions are conducive to the emergence of long-term persistence of infection in a population?* In order to address this question we will consider cellular-automata based descriptions of infection spreading, for a disease that has temporary immunity^11^,^12^. We will consider initial populations with varying degrees of global heterogeneity, reflecting increasing diversity in the condition of the individuals comprising the population. Our attempt will be to ascertain the influence of this heterogeneity on the persistence of infection. The model we will consider combines two distinct features. The first is the transition from the susceptible to the infected state, determined by the state of the immediate neighbourhood, which is *stochastic* in nature. The second feature is a *deterministic* disease cycle, which ensues upon infection. We give details below.

## Model

Mathematically, epidemiological models have successfully captured the dynamics of infectious disease^20^,^21^. One well known model for non-fatal communicable disease progression is the SIRS cycle. This model appropriately describes the progression of diseases with temporary immunity, such as small pox, tetanus, influenza, typhoid fever, cholera and tuberculosis^16^,^17^.

The SIRS cycle is described by the following disease compartments. At the outset an individual is *susceptible* to infection (a stage denoted by S). On being infected by contact with other infected people in the neighbourhood, the individual moves on to the *infectious* stage (denoted by I). This is followed by a *refractory* stage (denoted by R). In the refractory stage the individual becomes immune to disease and also does not infect others. But this immunity is temporary as the individual becomes susceptible again after some time interval.

Specifically, in this work we consider a cellular automata model of the SIRS cycle described above^11^,^12^,^13^. In this model of disease progression, time *t* evolves in discrete steps, with each individual, indexed by (*i, j*) on a 2 dimensional lattice, characterized by a counter


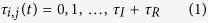


describing its *phase* in the cycle of the disease^11^. Here *τ*_*I*_ + *τ*_*R*_ = *τ*_0_, where *τ*_0_ signifies the total length of the disease cycle. At any instant of time *t*, if phase *τ*_*i,j*_(t) = 0, then the individual at site (*i, j*) is susceptible; if 1 ≤ *τ*_*i,j*_(*t*) ≤ *τ*_*I*_, then it is infected; if phase *τ*_*i,j*_(*t*) > *τ*_*I*_, it is in the refractory stage. For, phase *τ*_*i,j*_(*t*) ≠ 0 the dynamics is given by the counter updating by 1 every time step, and at the end of the refractory period the individual becomes susceptible again, which implies if *τ*_*i,j*_(*t*) = *τ*_0_ then, *τ*_*i,j*_(*t* + 1) = 0. Namely,


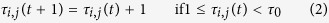



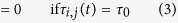


**Figure 1:**
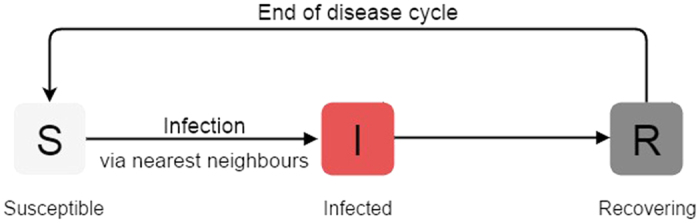
Schematic Representation of the SIRS cycle. The color scheme in all figures is as follows: black represents the refractory state (R); white represents the susceptible state (S); red represents the infected state (I).

Hence the disease progression is a *cycle* (see Fig. 1). We consider the typical condition where the refractory period is longer than the infective stage, i.e. *τ*_*R*_ > *τ*_*I*_.

We now investigate the spread of epidemic in a group of spatially distributed individuals, where at the individual level the disease progresses in accordance with the SIRS cycle. In particular, we consider a population of individuals on a 2-dimensional lattice where every node, representing the individual, has 4 neighbors^14^. Unlike many studies with periodic boundary conditions, here the boundaries are fixed with no interactions outside the boundaries. So our model mimics a patch of population, such as an island or an isolated habitat^3^, and investigates the persistence of infection in such a closed region.

### Condition for infection

Here we consider the condition that a susceptible individual (S) will become infected (I) *if one or more of its nearest neighbours are infected*. That is, if *τ*_*i,j*_(*t*) = 0, (namely, the individual is susceptible), then *τ*_*i,j*_(*t* + 1) = 1, if any 1 ≤ *τ*_*x,y*_(*t*) ≤ *τ*_*I*_ where *x, y* ∈ {(*i* − 1, *j*); (*i* + 1, *j*); (*i, j* − 1); (*i, j* + 1)}.

Notice that there are two distinct features determining the local state of the individuals. The first is the transition from the susceptible to the infected state determined by the state of the immediate neighbourhood, which is *stochastic* in nature and dependent on the distribution of initial states of the individuals in the population. The second feature is the *deterministic* disease cycle: I → R → S. This interplay of a probabilistic feature and a deterministic cycle shapes the dynamics of disease in the population^22^.

In this work we focus on an unexplored aspect of such systems, namely we attempt to ascertain the dependence of the persistence of infection on the *composition of the population*. So the specific question of relevance here will be the connection between sustained long-time persistence of infection in a region from an infective seed(s) and the heterogeneity of the states of the individuals in the region.

Heterogeneity, namely non-uniformity in the states of the individuals, may be characterized in different ways. Consider a generic initial population patch comprised of a random admixture of susceptibles, infected and refractory individuals, given by initial fractions *S*_0_, *I*_0_ and *R*_0_. So, if either *S*_0_, *I*_0_ or *R*_0_ tends to one, we have a homogeneous situation where almost all individuals are in the same state, namely almost all susceptible (*S*_0_ → 1), or almost all infected (*I*_0_ → 1), or almost all recovered (*R*_0_ → 1). Increasing deviations from this, reflects increasing heterogeneity in the population, as it implies an increasing spread among the different disease compartments.

Another source of heterogeneity arises from non-uniform stages of disease of the individuals within a disease compartment. In our study, we first consider the scenario where the sub-population of refractory individuals (given by *R*_0_) are in the same stage of the disease cycle, namely the heterogeneity is entirely reflected by the variation of *S*_0_, *I*_0_ and *R*_0_. We then go on to study the effect of non-uniformity in the stages of recovery of the refractory individuals, bringing in yet another type of heterogeneity in the population, reflecting the spread in the states of individuals in the same disease compartment.

In the sections below, we now present our simulation results, focussing on the persistence of infection, obtained by sampling a large range of initial states, reflecting varying degrees of heterogeneity, both in the partitioning of the population into disease compartments, as well as diversity in the disease phases within a compartment.

## Spatio-temporal patterns of infection spreading

We first focus on the infection spreading patterns in the population. The principal question we ask is the following: when is infection persistent in a patch, and how this depends on the constitution of the initial population. In order to examine this, we study the spread of infection from a seed of infection (namely one or two infected individuals) across a patch of population composed of individuals at different stages in the disease cycle, and with varying degrees of heterogeneity in the population.

With no loss of generality we consider *τ*_*I*_ = 4; *τ*_*R*_ = 9; *τ*_0_ = 13 and a lattice of size 100 × 100. In our figures we represent the state of an individual in the disease cycle (namely S, I or R) by a color, with white denoting a susceptible individual, black denoting a refractory individual and red denoting an infected individual. The fraction of susceptible individuals in the population at time *t* is denoted by *S*_*t*_, the fraction of infected individuals by *I*_*t*_ and the fraction of refractory individuals by *R*_*t*_. In the sections below we will focus on the possibility of the prolonged existence of infection arising in different classes of initial populations, characterized by different *S*_0_, *R*_0_ and *I*_0_. Namely, our central focus is to explore the probability with which a seed(s) of infection leads to persistant infection in the population.

## Non-persistent Infection in a Homogeneous Susceptible Population

**Figure 2:**
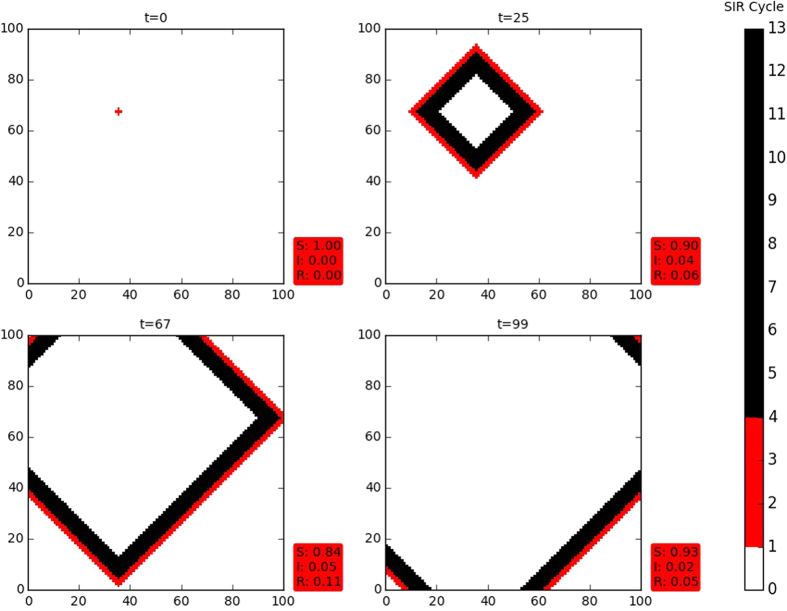
Snapshots at times *t* = 0, 25, 67, 99, showing the spread of infection from one infected individual at *t* = 0, in a homogeneous initial population comprising entirely of susceptible individuals (i.e. *S*_0_ ~ 1, *R*_0_ = 0, *I*_0_ ~ 0). The long bar shows the relative lengths of the susceptible (S), infected (I) and refractory (R) stages in the disease cycle, where *τ*_*I*_ = 4, *τ*_*R*_ = 9 and the total disease cycle *τ*_0_ is 13 (see text). The red box shows the fraction of S, I and R individuals in the population at that instant of time.

First we investigate the effect of an infected individual on a population patch where *all individuals are entirely susceptible to infection*. Namely, we consider the case where at the outset there is one infected individual and the rest of the population is in the susceptible state, with *τ*_*ij*_ = 0. Figure 2 displays the spreading patterns obtained in such a scenario. It is clearly evident that as time progresses the infection starts from the infected individual (“seed”) and spreads symmetrically. This symmetric spreading pattern is readily understood from the condition for infection, which turns susceptible individuals to infected if any one of their neighbors is infected. So the infected seed infects its four neighbors, and these newly infected individuals in turn infect their nearest four neighbours, and so on. This process leads to an isotropic wave of infection which stops at the boundaries. In contradistinction, periodic boundary conditions^9^, or the presence of non-local “short-cuts” in space^8^,^10^, would place the infecteds in the proximity of suscpetibles again, thereby perpetuating the infection.

We confirmed the generality of these observations for different relative lengths of the infectious and refractory periods, namely varying *τ*_*I*_ and *τ*_*R*_ (with *τ*_*I*_ < *τ*_*R*_). We further ascertained that the choice of the location of the infected individual did not affect these qualitative trends.

Now the key factor in infection spreading is the contact of susceptible individuals with infected ones. It is clear that such an interaction takes place only at the outer edge of the wave of infection, while the inner boundary of the infected zone is contiguous only to refractory individuals. So the infection only spreads outwards, and does not move back into the interior of the lattice again.

Importantly then, the infection is removed after a while from the closed region, and all the individuals (including the original infective seed) comes to the end of the disease cycle and becomes susceptible again. So there is no infective site left in the population to perpetuate the infection and initiate another wave of disease spreading. Thus a *fully susceptible population does not allow the infection to persist.*

## Persistent infection in Heterogeneous Populations

Next we investigate the infection spread in the more realistic scenario where both refractory (*τ*_*i,j*_ > *τ*_*I*_) and susceptible individuals (*τ*_*i,j*_ = 0) are present in the initial population, and are randomly distributed spatially. We first consider the case where the refractory individuals have phases *τ*_*i,j*_ = *τ*_*I*_ + 1, namely, they are at the start of the refractory stage of the disease cycle. We investigate the persistence of infection in heterogeneous populations, with the initial state having (a) a single seed of infection and (b) varying initial fractions of infected individuals (*I*_0_). In both scenarios, we analyze the effect of varying *S*_0_ and *R*_0_ on the persistence of infection.

**Figure 3:**
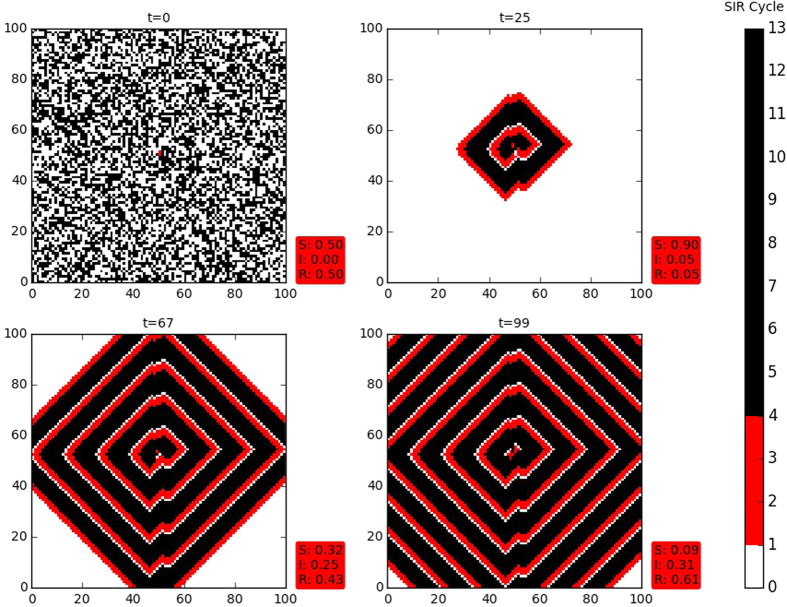
Snapshots of the infection spreading pattern in a heterogeneous population comprising initially of a random mixture of equal numbers of susceptible and refractory individuals (*S*_0_ ~ 0.5, *R*_0_ ~ 0.5 and *I*_0_ ~ 0), with one infected individual at *t* = 0. Here the refractory individuals have phases *τ*_*i,j*_ = *τ*_*I*_ + 1 (namely, they are at the start of the refractory stage of the disease cycle). Again, the long bar shows the relative lengths of the susceptible (S), infected (I) and refractory (R) stages in the disease cycle, where *τ*_*I*_ = 4, *τ*_*R*_ = 9 and the total disease cycle *τ*_0_ is 13 (see text). The red box shows the fraction of S, I and R individuals in the population at that instant of time. Interestingly, the spatially random population evolves into a more regular pattern after a short transient time.

To begin with, in Fig. 3, we illustrate the effect of a single infected individual on an initial population with equal numbers of susceptible and refractory individuals, namely *S*_0_ = *R*_0_. It is evident from these representative results that in a well mixed population, consisting of a random collection of both susceptible and refractory individuals, introduction of a single infected individual can lead to *persistent infection in the population.* Also notice that some of these spreading patterns are reminiscent of coalescing and interacting spiral waves initiated by local inhomogeneity in an uniform background^15^.

This can be rationalized as follows: the mixed presence of susceptible and refractory individuals, implies that the disease cycles of the individuals in the population are *not synchronized*^19^. So there are always some individuals in the infective stage of the disease cycle in the population, and these act as seeds for continued infection propagation, leading to persistent infection. *Counter-intuitively then, the presence of individuals who are (temporarily) immune to the disease amongst susceptible ones leads to sustained infection, while in a completely susceptible population the infection dies out.*

**Figure 4:**
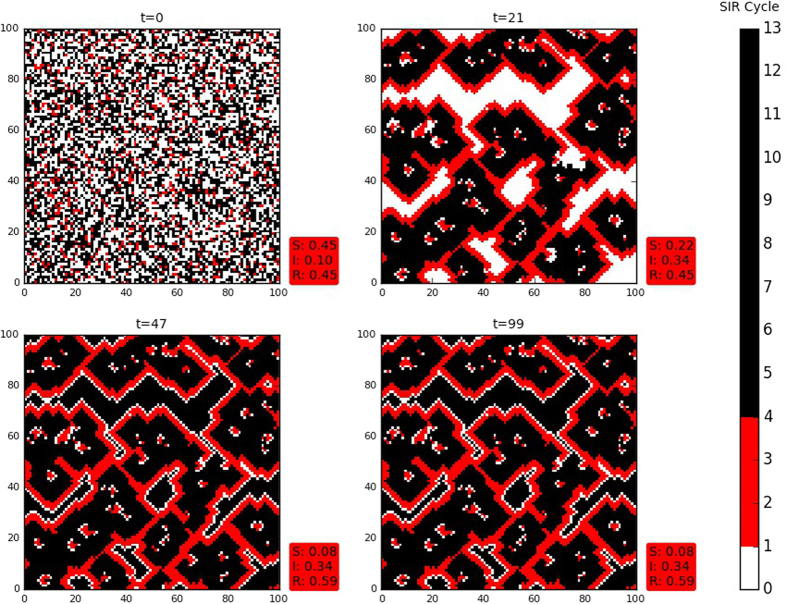
Snapshots of the infection spreading pattern in a heterogeneous population comprising initially of a random mixture of individuals, with *S*_0_ = *R*_0_ and *I*_0_ = 0.1. Here the refractory individuals have phases *τ*_*i,j*_ = *τ*_*I*_ + 1 (namely, they are at the start of the refractory stage of the disease cycle). Again, the long bar shows the relative lengths of the susceptible (S), infected (I) and refractory (R) stages in the disease cycle, where *τ*_*I*_ = 4, *τ*_*R*_ = 9 and the total disease cycle *τ*_0_ is 13 (see text). The red box shows the fraction of S, I and R individuals in the population at that instant of time.

Next we focus on the time evolution of an initial population consisting of a random mixture of *S, I* and *R* states. In particular we investigate the nature of the persistent infection in the population under varying initial fractions of infected individuals *I*_0_. A typical random initial condition is shown in Fig. 4, with the initial fraction of infected sites *I*_0_ being one-tenth and the initial fraction of susceptibile and refractory individuals being equal (i.e. *S*_0_ = *R*_0_). Here too we find that infection is sustained.

**Figure 5:**
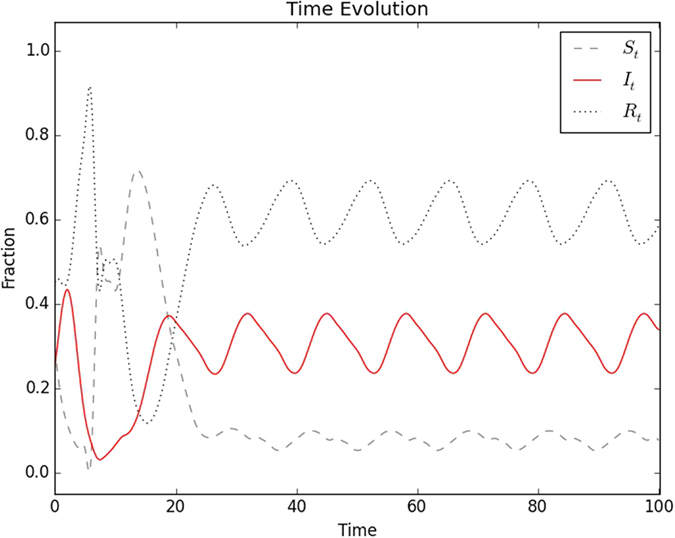
Time evolution of *I*_*t*_, *S*_*t*_, *R*_*t*_, in a heterogeneous population comprising initially of a random mixture of individuals, with *S*_0_ = *R*_0_ and *I*_0_ = 0.1.

Further, interestingly, it is clear that there is an *approximate recurrence of the complex patterns of infected individuals in the population*. Figure 5 shows the time evolution of the fraction of infected, refractory and susceptible individuals in the population, namely *I*_*t*_, *R*_*t*_ and *S*_*t*_, in the case displayed in Fig. 4. It can be clearly seen that after transience, *I*_*t*_, *R*_*t*_ and *S*_*t*_ exhibit steady oscillatory dynamics, with period of oscillation close to the disease cycle length *τ*_0_. This is consistent with the observed recurrence of the spatio-temporal patterns when persistent infection emerges.

**Figure 6:**
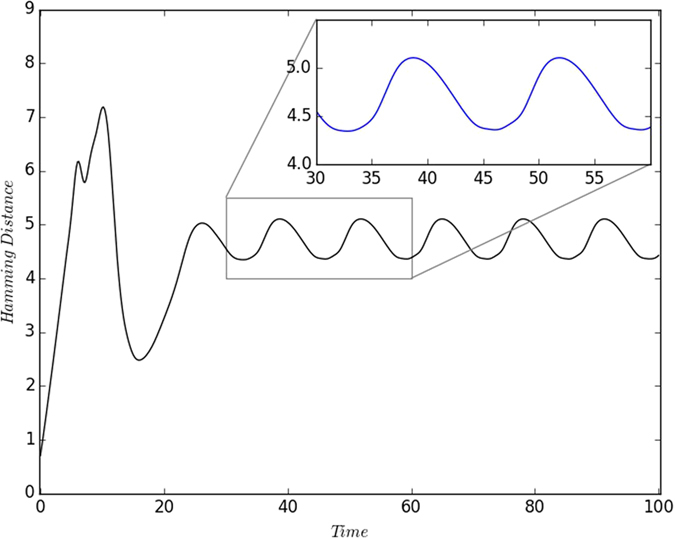
Hamming distance given by Eqn. 4 as a function of time, in a heterogeneous population comprising initially of a random mixture of individuals, with *S*_0_ = *R*_0_ and *I*_0_ = 0.1.

A quantitative measure of the recurrence of patterns can also be obtained by calculating the difference of the state of the population from the initial state, as reflected by the Hamming distance:


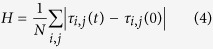


where the sum is over all *N* sites in the lattice. The time dependence of the Hamming distance given above is shown in Fig. 6, and it clearly shows steady oscillations. This indicates that the fraction of the susceptible, infected and refractory individuals in the population, and more remarkably their *locations*, repeat almost periodically over time. It should be noted that the frequency of oscillation again approximately corresponds to the disease cycle length.

Another pertinent observation here is the dependence of this dynamics on disease cycle. As the length of the infectious stage (i.e. *τ*_*I*_) increases, keeping the total disease cycle length invariant, the fraction of infected individuals *I*_*t*_ increases. The average *I*_*t*_ is proportional to the fraction of the disease cycle occupied by the infectious stage, i.e the ratio *τ*_*I*_/*τ*_0_. So the size of the infected population strongly depends on the nature of disease as reflected in the length of the infectious stage of the disease.

## Influence of the initial composition of the population on the persistence of infection

We now attempt to gauge the statistically significant trends in *I*_*t*_, by averaging the fraction of infected individuals at asymptotic time *t*, arising from a wide range of random initial configurations at time *t* = 0. We denote this by 〈*I*_*t*_〉. Such a global measure provides a quantitative estimate of the size of the basin of attraction of the persistent state.

In terms of this quantity, persistent infection is indicated by a non-zero value. However, after sufficient transient timesteps, if 〈*I*_*t*_〉 is zero, it indicates that the infection has died out. So 〈*I*_*t*_〉 can serve as an order parameter for the transition to sustained infection in a population.

## Dependence of persistence of infection on the initial fraction of susceptibles

For fixed *τ*_*I*_ and *τ*_0_ we have calculated 〈*I*_*t*_〉, for different initial fractions of susceptible individuals *S*_0_. We explore the full possible range of *S*_0_ ∈ [0, 1], where *S*_0_ = 0 signifies a population comprised entirely of refractory individuals who are immune to infection initially, and *S*_0_ = 1 implies an initial population comprised entirely of individuals susceptible to infection. While the phase of the susceptible (S) sub-population is *τ*_*i,j*_ = 0 of course, the refractory individuals (R) can be present in different stages in the refractory period with *τ*_*I*_ < *τ*_*i,j*_ < *τ*_0_. We explore two different scenarios of the initial state of the refractory individuals in the population.

**Figure 7:**
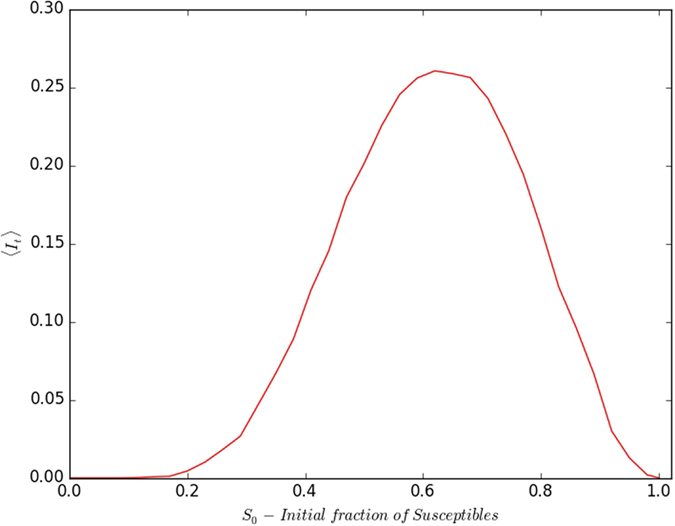
Variation of 〈*I*_*t*_〉 (after transience) with respect to the fraction of susceptible individuals in the initial population *S*_0_, arising from the presence of a single infected individual at time *t* = 0. Here the refractory individuals have the same phase, the disease cycle has *τ*_*I*_ = 4; *τ*_0_ = 13, and *I*_*t*_ is averaged over 10^3^ realizations of the initial population on the lattice. The specific case of a 100 × 100 lattice is displayed. However note that different lattice sizes yield the same result.

First we present the case where all the refractory individuals are at the start of the refractory stage of the disease cycle, i.e. all *τ*_*i,j*_ = *τ*_*I*_ + 1. So there is uniformity in the stage of disease progression in the refractory sub-population, though the individuals are randomly distributed spatially. We focus on the asymptotic state of infection in such a population, arising from a single infected individual at the outset. The results obtained from a large sample of initial states is shown in Fig. 7, and it is evident from there that 〈*I*_*t*_〉 is *very low for both high and low S*_0_, peaking around *S*_0_ ~ 0.65–0.75. Namely, homogeneous initial populations where all individuals are immune (*S*_0_ = 0), or all are susceptible to disease (*S*_0_ = 1), do not yield persistent infection. Rather, mixed populations lead to most sustained infection, with persistently high numbers of infected individuals.

We can rationalize our observations as follows: If an infected individual is completely surrounded by refractory individuals with *τ* = *τ*_*I*_ + 1, it will complete the infectious stage without transferring the infection at all, as *τ*_*I*_ < *τ*_*R*_. So the infection can spread only if the infected seed is contiguous to at least one susceptible individual. Now the probability of contact with a susceptible individual in the initial stages of infection spreading depends on the initial fraction of susceptibles *S*_0_. This suggests that when *S*_0_ is low, the chance of the infected individual being in contact with a susceptible one is low. As a result, as *S*_0_ tends to zero, on an average, the infection eventually gets removed from the population, with the seed of infection crossing over to the refractory phase without infecting any other individual.

When there are more susceptible individuals in the initial population, there is a higher chance that the infected seed will encounter a susceptible neighbour. So as expected, increasing *S*_0_ leads to a larger infected set on an average. However the surprising trend is the *decrease* in the infected set as the initial susceptible sub-population becomes too high, with the number of infected individuals tending to zero as the entire population becomes susceptible. This feels counter-intuitive, but can be understood as follows: Consider the limiting case where initially almost all the individuals are susceptible to the infection. Now the infection will spread immediately in isotropic waves, but will eventually stop at the boundaries. In analogy to the spread of forest fire, the boundary of refractory individuals is like scorched earth preventing spread across them. Now after the wave of infection passes, the individuals are in the refractory stage, leading eventually to the entire set being synchronized in the susceptible regime. There is no infected individual left then to act as a seed for a further wave of infection spreading. So the infection does not persist. The susceptible stage is like an “absorbing state”, and in the absence of “infectious perturbation” the system remains fixed in that state.

In order to prevent the above scenario, one needs enough refractory individuals in the population. When *R*_0_ is below 1/4 (i.e. *S*_0_ > 3/4), typically the infected seed may not have a refractory individual among its four neighbours. So one expects that the persisting infection will have lower probability of occurrence as *S*_0_ increases beyond 3/4. This is in accordance with the trends observed in the simulations.

We then see that for the *infection to persist* in a population, a *well mixed heterogeneous population is required*, with reasonable number of both susceptible and refractory individuals. *Randomly mixed populations prevent synchronization of the disease*, and this is the key to always having some source of infection left in the population.

## Dependence of persistence of infection on the initial fraction of infecteds

**Figure 8:**
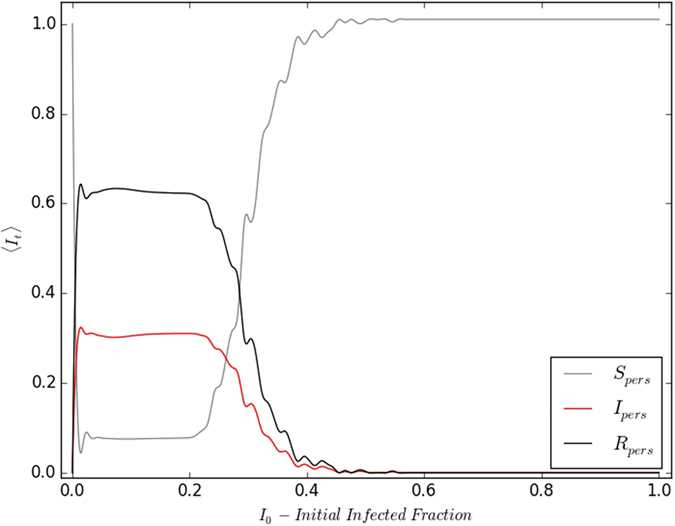
Variation of 〈*I*_*t*_〉 (after transience) with respect to the initial fraction of infected individuals *I*_0_ in the population, and *S*_0_ = *R*_0_. The refractory sub-population consists of individuals with phase equal to *τ*_*I*_ + 1. Here the disease cycle has *τ*_*I*_ = 4; *τ*_0_ = 13, and *I*_*t*_ is averaged over 10^3^ initial realizations. The specific case of a 100 × 100 lattice is displayed. However note that different lattices sizes yield the same result.

We now vary the initial fraction of infected individuals *I*_0_ in the population, over the entire range [0, 1]. For the remaining population, the initial fraction of susceptible and refractory individuals is set at different ratios. We consider an ensemble of initial conditions, with specific *I*_0_, *S*_0_ and *R*_0_ and find the time averaged *I*_*t*_, after long transience for each realization. The ensemble average of this quantity is displayed in Fig. 8. Notably, we find that there is a definite *window of persistence* over the range of *I*_0_, where the infection never dies down and the fraction of infected individuals in the population is reasonably high on an average.

In the state where infection is persistent, the individuals are unsynchronized and spread over the different stages of the disease cycle. So on an average the fraction of infected individuals is ~*τ*_*I*_/*τ*_0_, namely the fraction of the total disease cycle occupied by the infected stage. For instance, in the example shown in Fig. 8 with *τ*_*I*_ = 4 and *τ*_0_ = 13, at the plateau of persistence, the infected fraction is approximately one-third of the population.

The transition to persistent infection is sharp and occurs at *I*_0_ → 0. This implies that *the infection can spread and persist even when there is only a single infected individual in the initial population*. This is consistent with the results we presented earlier (cf. Fig. 7) on infection spreading from a single infected individual.

Interestingly, the infection ceases to persist for higher values of *I*_0_, and the fall in persistence is rapid. That is, if the initial population has too many infected individuals, infection will not persist. This can be rationalized by noting that one needs a mix of susceptibles and refractory individuals in the population for persistent infection. For instance, considering the limiting case of all infected individuals in the initial state, it is clear that all individuals will go through the disease cycle in synchrony. So all individuals will become susceptible again together, but there will be no infective seed left in the population to perpetuate the infection.

## Effect of varying degrees of non-uniformity in the refractory sub-population on the persistence of infection

Now we will explore the effect of non-uniformity within the refractory sub-population on the emergence of persistent infections. Namely, we will consider the refractory individuals in the initial population to be in different stages of disease progression. We will consider two distinct ways of interpolating between the completely heterogeneous and completely uniform limiting cases, in order to gauge the effect of heterogeneity on sustaining infection.

First we consider the initial refractory sub-population to be an admixture of subsets of individuals with uniform phase and with randomly distributed phases. Specifically, we explore initial refractory sub-populations comprised of some fraction *f*_*rand*_ with phases randomly distributed over the range *τ*_*I*_ + 1 to *τ*_0_, and the rest 1 − *f*_*rand*_ with fixed phase *τ*_*R*_ = *τ*_*I*_ + 1. We examine the spread and persistence of infection in such a scenario, under variation of the initial composition of the population.

**Figure 9:**
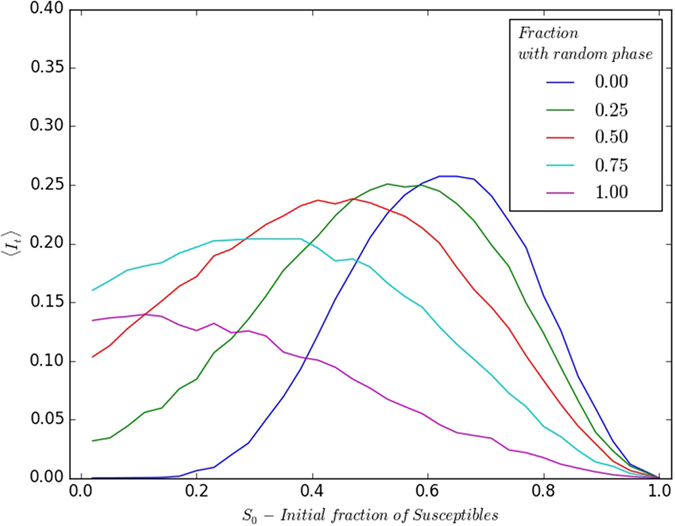
Variation of 〈*I*_*t*_〉 (after transience) with respect to initial fraction of susceptible individuals *S*_0_, for different fractions *f*_*rand*_ of the initial refractory sub-population having randomly distributed phases (see key). Here the disease cycle has *τ*_*I*_ = 4; *τ*_0_ = 13, and *I*_*t*_ is averaged over 10^3^ initial realizations and lattice size is 100 × 100.

Figure 9 exhibits the persistence of infection, with respect to varying *S*_0_, arising in a population that had a single infected individual initially. Different fractions of the initial refractory sub-population with randomized phases were explored, ranging from *f*_*rand*_ = 0 (i.e. completely uniform), to *f*_*rand*_ = 1 (i.e. completely heterogeneous). The trends clearly indicate a continuous cross-over from the condition where all refractory individuals are in the same phase, to the scenario where all are in random phases.

**Figure 10:**
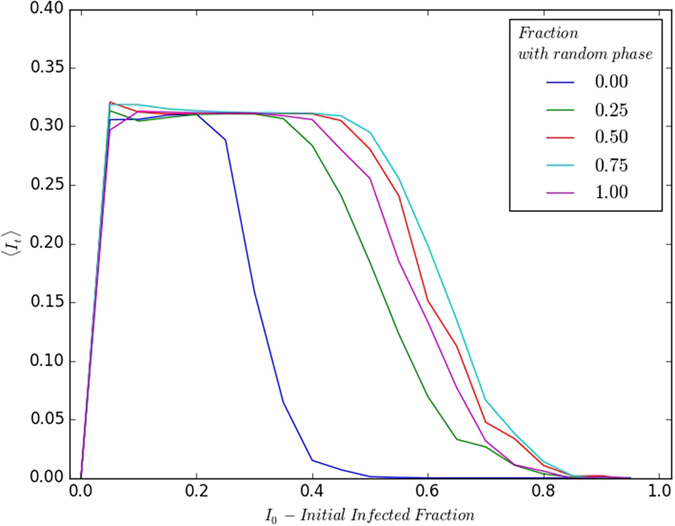
Variation of 〈*I*_*t*_〉 (after transience) with respect to the initial fraction of infected individuals *I*_0_ in the population, and *S*_0_ = *R*_0_. The initial refractory sub-population consists of different fractions *f*_*rand*_ with randomly distributed phases (see key). Here the disease cycle has *τ*_*I*_ = 4; *τ*_0_ = 13, and *I*_*t*_ is averaged over 10^3^ initial realizations. While the specific case of a 100 × 100 lattice is displayed, different lattices sizes yield the same result.

Further, we explore the effect of varying the initial fraction of infected individuals *I*_0_, over the range [0, 1]. Figure 10 exhibits the change in the window of persistence with respect to *f*_*rand*_. It is evident that increasing *f*_*rand*_, namely increasing the initial number of refractory individuals with *de-synchronized phases*, leads to a definite increase in the window of persistence. This implies that *for populations with a more heterogeneous refractory sub-population, the disease persists over a larger range of infected fractions I*_0_ *of the initial population*.

Note however, that there is also an apparent reduction in the window of persistence at very high *f*_*rand*_. This can be rationalized by noting that when the entire initial refractory sub-population *R*_0_ has uniformly distributed phases, there are a significant number of individuals who are closer to the end of their disease cycle (for instance, stage 12 or 13). These individuals become susceptible within a few time steps, and therefore bring the population closer to an overall state of homogeneity again, as all susceptibles are in the same phase (stage 0) and remain in that phase unless infected. We have observed qualitatively and quantitatively earlier, that a more homogeneous population leads to a reduced window of persistence. Hence, *presence of a significant number of individuals closer to the end of their disease cycle acts as a homogenizing factor for the population and is detrimental to persistence*.

Lastly, we study the effect of varying ranges of spread in the initial phases of the refractory individuals. Specifically we consider that the phase of the refractory individuals in the initial population to be randomly distributed over different ranges *R*_*rand*_. In particular we examine the persistence of infection for *R*_*rand*_ ranging from [*τ*_*I*_ + 1,*τ*_*I*_ + 1], (where all refractory individuals have the same phase) to [*τ*_*I*_ + 1, *τ*_*I*_ + *τ*_*R*_] (where heterogeneity is large as the phases of the refractory individuals are distributed over the entire refractory range).

**Figure 11:**
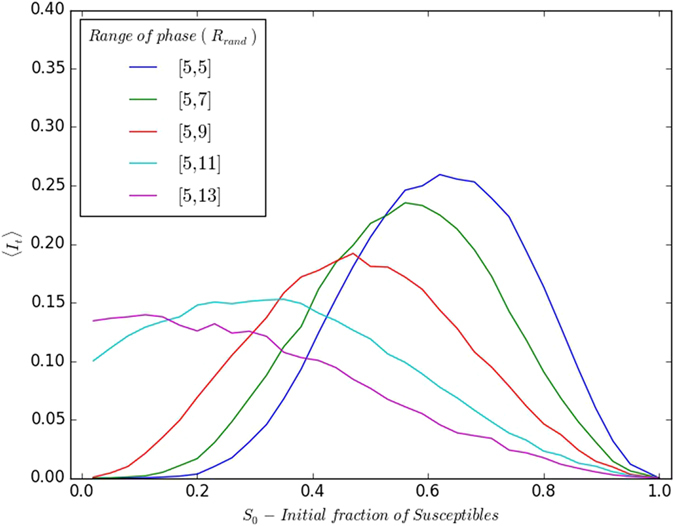
Variation of 〈*I*_*t*_〉 (after transience) with respect to initial fraction of susceptible individuals *S*_0_, for the case where there is a *single* infected individual in the population at the outset, and the refractory individuals in the population have phases *τ* randomly distributed over different ranges *R*_*rand*_ in the refractory stage: [5,5]; [5,7]; [5,9]; [5,11]; [5,13]. Here *I*_*t*_ is averaged over 10^3^ realizations, lattice size is 100 × 100, and the disease cycle parameters *τ*_*I*_ = 4, *τ*_0_ = 13.

**Figure 12:**
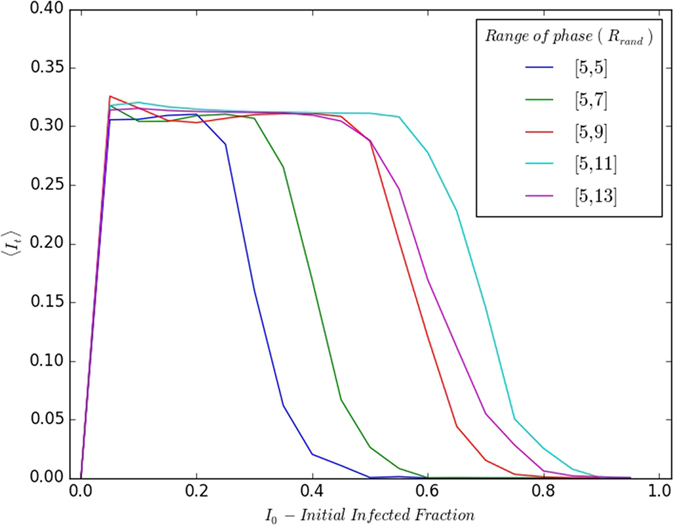
Variation of 〈*I*_*t*_〉 (after transience) with respect to initial fraction of infected individuals *I*_0_, for the refractory individuals having phases *τ* randomly distributed over different ranges *R*_*rand*_ in the refractory stage: [5,5]; [5,7]; [5,9]; [5,11]; [5,13]. Here *I*_*t*_ is averaged over 10^3^ realizations, lattice size is 100 × 100, and the disease cycle parameters *τ*_*I*_ = 4, *τ*_0_ = 13.

Figures 11 and 12 exhibit representative results of 〈*I*_*t*_〉 as a function of the initial fraction of susceptibles *S*_0_ and infecteds *I*_0_. It can be clearly seen that a smooth cross-over takes place from the extremal case of all refractory individuals in the same phase, to the limit where the stages of the refractory individuals are spread randomly over the entire refractory period. The key observation here is that as the spread in phases increases, the range of persistent infection becomes larger. Namely, when there is a large initial spread in the stages of disease among the individuals, at subsequent times there are always some individuals who can “pick up the baton of infection”, leading to persistent infection.

So we see that in the completely heterogeneous case, low susceptible and high refractory initial subpopulations favour persistent infection. But in a completely uniform population, a higher fraction of susceptibles leads to persistent infection. This has the following important implication: when refractory individuals are not synchronized at the same phase of disease progression, even if there are few susceptible individuals in the population initially, the infection grows substantially and the average size of the infected sub-population is large. So we have demonstrated that even when the entire population is susceptible to infection, the infection eventually dies out, while even a few susceptibles among an heterogeneous refractory population gives rise to a large persistent infected sub-population.

We can rationalize this counter-intuitive trend that persistent infection is more likely when the number of susceptible individuals in the initial population is low, as follows: When *S*_0_ is low, there are many refractory individuals in the population surrounding the infected individual. These individuals are in various stages in the refractory period, and some become susceptible again while the seed is still infectious. If *S*_0_ → 0 and the refractory individuals are uniformly distributed over the refractory range *τ*_*R*_, the probability of the seed encountering a susceptible individual while still infectious is proportional to *τ*_*I*_/*τ*_*R*_. Since at least one neighbour in contact with the seed needs to be susceptible, this probability should be greater than 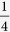 for the infection to spread, on an average. So when the infective stage *τ*_*I*_ is sufficiently long (as in our example of *τ*_*I*_ = 4, in a disease cycle of length 13), extremely low initial *S*_0_ can also lead to persistent infection.

Lastly, note that certain systems in the broad class considered here, have found persistent infection arising due to the presence of “short-cuts” or non-local connections in space. Such long-range links allow a distant infectee to jump through to re-infect and enable perpetuatuation^8^,^10^. However, in our case there are no such “short-cuts” aiding persistence. Rather persistence of infection arises from the initial heterogeneity of the population, and this suggests yet another origin of self-sustained infection in a population.

## Discussion

In summary, we have explored infection spreading qualitatively and quantitatively in a patch of population, where the disease progression of the individuals was given by the SIRS model and an individual became infected on contact with another infected individual. Such an island or isolated patch or habitat, can provide a “natural laboratory” to study spread of epidemics^3^. Here we have focussed on the emergence of peristent infection in the patch, under varying degrees of heterogeneity in the initial population.

Specifically, we considered two scenarios of diversity in the population. In one we considered varying fractions of the initial population in different disease compartments, and in another we examined varying spread in the phases of disease progression among the individuals. Our central result is the following: we find that an infectious seed does not give rise to persistent infection in a homogeneous population consisting of individuals at the same stage of disease progression. Rather, when the population consists of randomly distributed individuals at various stages of the disease, infection becomes persistent in the population patch.

Now the initial state of the patch of population can occur in different ways. First, a random set of individuals may have colonized the patch, and so the initial state is typically a random mix of individuals. Alternately, we can think of a population comprised of individuals susceptible to a disease being invaded by individuals that may be infected or recovering (refractory), namely a small set of infective or recovered individuals, enter an island/patch of population comprised of individuals susceptible to the disease. The question then is, will this entry lead to persistent infection in the patch? The interesting indication of our study is that if only infecteds enter a population that is entirely made of susceptibles, the infection will die out. However, if a few infecteds (even one) enters along with some refractory individuals the infection will persist. Alternately, if susceptible and refractory individuals are quarantined in a patch, a single infected can lead to sustained infection. Further, if individuals who have recovered from the disease at different points in time are in an isolated group, the entry of even a single infected individual can lead to persistent infection in the group, while the entry of an infected in a group of entirely susceptible individuals or individuals at the exact same stage of recovery, will only lead to transient waves of infection, which will soon die out.

In order to broadly gauge the underlying mechanism that leads to the persistence of infection, one must actually focus on the scenarios where infection burns out. Infections die out eventually when there is too much synchrony in the population, as this can lead to most of the patch entering the refractory stage, and subsequently the susceptible stage, simultaneously. This leaves no infective seed in the population, and no new wave of infection can be initiated. For persistence of infection one then needs a *balance* between sufficiently large number of susceptibles (so that the disease can spread), as well as enough refractory individuals (so that there is no synchronization). Namely, an admixture of susceptibles and refractory individuals in the immediate neighbourhood of an infected allows spread of disease, without the entire neighbourhood entering the infective stage synchronously. The counter-intuitive consequence of this is that infection eventually dies out when an infective seed enters a population that is entirely susceptible, while its entry in a population comprised of individuals in different stages of recovery (some of whom will become susceptible within the infective period of the infected individual) gives rise to persistent infection. So our observations suggest that initial heterogeneity leads to greater propensity for sustaining an infected sub-population, thereby facilitating persistent infection.

## Additional Information

**How to cite this article:** Agrawal, V. *et al*. Emergence of Persistent Infection due to Heterogeneity. *Sci. Rep.* **7**, 41582; doi: 10.1038/srep41582 (2017).

**Publisher's note:** Springer Nature remains neutral with regard to jurisdictional claims in published maps and institutional affiliations.
